# Acceleration of Wound Healing by α-gal Nanoparticles Interacting with the Natural Anti-Gal Antibody

**DOI:** 10.1155/2015/589648

**Published:** 2015-04-01

**Authors:** Uri Galili

**Affiliations:** Department of Surgery, University of Massachusetts Medical School, Worcester, MA 01655, USA

## Abstract

Application of *α*-gal nanoparticles to wounds and burns induces accelerated healing by harnessing the natural anti-Gal antibody which constitutes ~1% of human immunoglobulins. *α*-gal nanoparticles present multiple *α*-gal epitopes (Gal*α*1-3Gal*β*1-4GlcNAc-R), the carbohydrate ligand of anti-Gal. Studied *α*-gal nanoparticles were comprised of glycolipids with *α*-gal epitopes, phospholipids, and cholesterol. Binding of anti-Gal to *α*-gal nanoparticles in wounds activates the complement cascade, resulting in formation of chemotactic complement cleavage peptides that induce rapid recruitment of many macrophages. The Fc/Fc*γ* receptors interaction between anti-Gal coating *α*-gal nanoparticles and the recruited macrophages activates macrophages to produce cytokines/growth factors that promote wound healing and recruit stem cells. Studies of wound healing by *α*-gal nanoparticles were feasible in *α*1,3galactosyltransferase knockout mice and pigs. In contrast to other nonprimate mammals, these mice and pigs lack the *α*-gal epitope, and thus they are not immunotolerant to it and produce anti-Gal. Treatment of skin wounds and burns with *α*-gal nanoparticles resulted in 40–60% decrease in healing time in comparison with control wounds treated with saline. This accelerated healing is associated with increased recruitment of macrophages and extensive angiogenesis in wounds, faster regrowth of epidermis, and regeneration of the dermis. The accelerated healing further decreases and may completely eliminate fibrosis and scar formation in wounds. Since healing of internal injuries is mediated by mechanisms similar to those in external wound healing, it is suggested that *α*-gal nanoparticles treatment may also improve regeneration and restoration of biological function following internal injuries such as surgical incisions, myocardial ischemia following infarction, and nerve injuries.

## 1. Significance of Macrophages in Wound Healing

Wounds that penetrate deep in the skin remain a major clinical problem because of morbidity associated with prolonged periods required for repair and regeneration of the injured tissue, bleeding, risk for infections and septicemias, keloids, and scar formation. These risk factors are further exacerbated in patients with impaired wound healing as in diabetes and in old age. Accelerating wound healing process can minimize these risk factors. Moreover, because of the increase in the proportion of aging individuals in the population in the coming decade, the costs of clinical care for wound healing are likely to greatly increase [1].

Healing of acute wounds requires the local effective recruitment and activation of macrophages which are the pivotal cells in early stages of injury healing. After debriding the injured tissue by phagocytosis, macrophages undergo transition into “prohealing” cells that mediate repair and regeneration by secreting cytokines/growth factors that induce regeneration of epidermis, dermis, and capillary network (angiogenesis) [2–9]. Macrophages originate from blood monocytes that migrate into the wound in response to monocytes chemoattractant cytokines such as MCP-1 (monocyte chemoattractant protein-1), MIP-1 (macrophages inflammatory protein 1), and RANTES (regulated on activation, normal T cell expressed and secreted) released from cells within and around injury sites [10–15]. Whereas small wounds may be completely healed and the injured tissue regenerated within several days, the healing process of the large wounds or burns may be much longer. A prolonged healing increases the risk of infection and increases the probability of scar formation and prevention of remodeling of the injured tissue into its original structure. Scar formation is the default mechanism for repair of injuries. In scar formation, a dense connective tissue (fibrosis) replaces the original structure of the injured tissue. The longer the healing time of a wound, the higher the probability that a scar will be formed. Based on these considerations, it is reasonable to assume that rapid recruitment of macrophages into wounds may decrease morbidity, including the risk of infection, and reduce the extent of scar formation, or completely avoid this default repair mechanism.

A very effective physiologic mechanism for inducing rapid recruitment of macrophages into wounds is the activation of the complement system within injured tissues by antigen/antibody (Ag/Ab) interaction. Such activation results in generation of chemotactic complement cleavage peptides, including C5a and C3a which induce vasodilation, extravasation of blood monocytes, maturation of these monocytes into macrophages, and their migration along the complement chemotactic gradient into the complement activation site. This mechanism is of major significance in microbial infections where Ab binding to microbial Ags activates the complement system and generates chemotactic complement cleavage peptides that induce rapid recruitment of neutrophils and macrophages into the infection site [16–18]. Rapid recruitment of macrophages into various injuries is feasible by harnessing the immunologic potential of the natural anti-Gal Ab [19–21]. This Ab is present in high titers in all humans that are not severely immunocompromised [22]. Interaction of anti-Gal with *α*-gal nanoparticles [23] within injuries results in effective activation of the complement system, recruitment of macrophages, and activation of the recruited macrophages to produce “prohealing” cytokines/growth factors that decrease healing time of injuries by 40–60% [19–21]. The accelerated wound healing process further decreases or completely prevents scar formation [19]. This review describes the natural anti-Gal Ab, *α*-gal nanoparticles, and experimental models in which anti-Gal/*α*-gal nanoparticles interaction accelerates wound healing. The review further discusses the possible use of *α*-gal nanoparticles in treatment of various internal injuries.

## 2. The Natural Anti-Gal Ab and Its Ligand, the ***α***-gal Epitope

Anti-Gal is a natural Ab produced in humans throughout life as ~1% of circulating immunoglobulins [22] and is found in the blood as IgG, IgM, and IgA classes [22, 24–27]. This Ab displays characteristics similar to anti-blood group A and anti-blood group B natural Abs in that its ligand is also a carbohydrate Ag called the *α*-gal epitope, with the structure Gal*α*1-3Gal*β*1-4GlcNAc-R [28]. In addition, like these anti-blood group Abs, anti-Gal is produced in response to continuous antigenic stimulation by bacteria of the normal gastrointestinal flora that present carbohydrate Ags with structures similar to the *α*-gal epitope [29]. However, in contrast to anti-A and anti-B Abs which are produced according to the blood type of each individual, anti-Gal is naturally produced in all humans. As many as 1% of circulating B cells in humans is capable of producing anti-Gal following immortalization by Epstein Barr virus (EBV), only ~0.2% of EBV immortalized B cells produces anti-A or anti-B Abs [30]. The majority of anti-Gal B cells in the body are quiescent, whereas those along the gastrointestinal track produce anti-Gal following antigenic stimulation by gastrointestinal bacteria. Complement mediated lysis following anti-Gal binding to *α*-gal epitopes has been observed with various nucleated cells [31–33] and with enveloped viruses presenting this epitope [34–36].

Anti-Gal and the *α*-gal epitope display a unique distribution in mammals. All nonprimate mammals tested (e.g., mice, rats, rabbits, dogs, pigs, etc.) as well as prosimians (lemurs) and New World monkeys (monkeys of South America) lack the anti-Gal Ab, but all produce its ligand the *α*-gal epitope as ~10^5^–10^7^ epitopes/cell [37, 38]. In contrast, Old World monkeys (monkeys of Asia and Africa), apes and humans lack the *α*-gal epitope because they lack the glycosylation enzyme *α*1,3galactosyltransferase (*α*1,3GT) synthesizing this epitope, but they all produce the natural anti-Gal Ab [22, 27, 37, 38]. Because of this reciprocal distribution of anti-Gal and the *α*-gal epitope in mammals, xenotransplantation of pig organs in humans or in Old World monkeys results in rapid (hyperacute) rejection of the xenograft [39–42]. The binding of anti-Gal of the xenograft recipient to the multiple *α*-gal epitopes on the xenograft endothelial cells induces complement activation and complement mediated cytolysis of these cells, resulting in rapid collapse of the vascular bed and rejection of the graft [40–42]. This complement mediated hyperacute rejection of xenografts is an in vivo manifestation of the effective complement activation by the natural anti-Gal Ab.

In addition to induction of complement mediated cytolysis, anti-Gal interaction with glycoproteins, viruses, or cells presenting *α*-gal epitopes opsonizes them and induces their effective uptake by various cells with Fc*γ* receptors (Fc*γ*R) including macrophages, dendritic cells, and NK cells [39, 43, 44]. It is further probable that deposits of the complement molecule C3b on cells or on particles binding anti-Gal also mediate uptake of various anti-Gal opsonized cells or particles into macrophages by interaction with C3b receptors (C3bR, also referred to as CR1 and CD35) on these cells.

The ubiquitous presence of anti-Gal in large amounts in all humans who are not severely immunocompromised provides an opportunity for harnessing this Ab for various therapies. In previous studies in *α*1,3GT knockout mice, anti-Gal was shown to enable the induction of a protective antitumor immune response by in vivo targeting tumor cells engineered to present *α*-gal epitopes to antigen presenting cells (APC) [45–47]. Similarly, viral vaccines presenting *α*-gal epitopes and immunocomplexed in vivo with anti-Gal were effectively targeted to APC and thus increased their immunogenicity by 10–100-fold [48, 49]. As described below, anti-Gal mediated complement activation and opsonization for Fc*γ*R mediated uptake by macrophages further accelerate wound healing following treatment of wounds with *α*-gal nanoparticles.

## 3. ***α***-gal Nanoparticles Interaction with the Anti-Gal Ab

**Figure 1:**
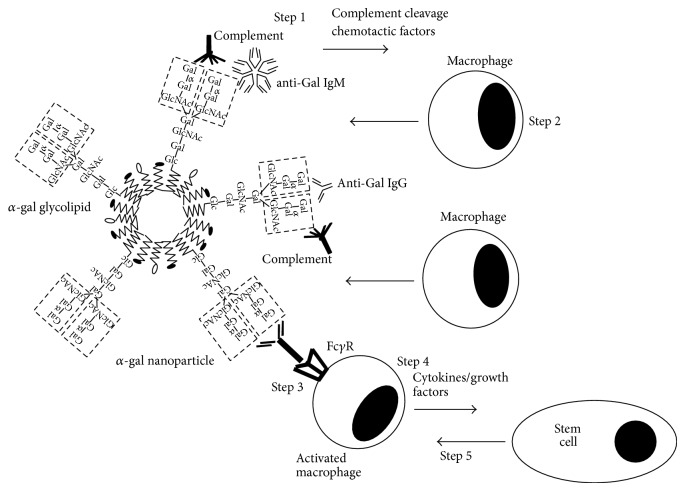
Illustration of *α*-gal nanoparticle and the process of accelerated wound healing induced by these nanoparticles. The studied *α*-gal nanoparticles are comprised of phospholipids, cholesterol, and *α*-gal glycolipids that carry *α*-gal epitopes (a representative 10 carbohydrate glycolipid with two branches, each capped with an *α*-gal epitope, marked by a dashed line rectangle). Application of *α*-gal nanoparticles to injuries results in the induction of the following sequential steps. (1) The natural anti-Gal Ab binds to *α*-gal epitopes on the nanoparticles and activates the complement system, resulting in formation of complement cleavage chemotactic peptides. (2) The chemotactic complement peptides induce rapid and extensive recruitment of macrophages into the treated injury. (3) The *α*-gal nanoparticles activate the recruited macrophages as a result of interaction between the Fc portion of anti-Gal coating the nanoparticles and Fc*γ* receptors (Fc*γ*R) on macrophages. (4) The activated macrophages produce “prohealing” cytokines/growth factors. (5) Secreted cytokines/growth factors further mediate rapid recruitment of stem cells into the injury site. Modified from [21].

*α*-gal nanoparticles are submicroscopic particles presenting multiple *α*-gal epitopes. Such nanoparticles may be prepared from various biodegradable materials. In studies on the effect of *α*-gal nanoparticles on wound healing, the nanoparticles with size range of 30–300 nm were prepared from glycolipids with multiple *α*-gal epitopes (*α*-gal glycolipids), phospholipids, and cholesterol (Figure 1) [19, 21]. These materials were obtained from chloroform: methanol extracts of rabbit RBC membranes. These RBC are suitable for the purpose of preparation of *α*-gal nanoparticles since they present the highest concentration of *α*-gal epitopes among mammalian RBC and since most of the glycolipids in their membranes carry *α*-gal epitopes [37, 47, 50–55]. Rabbit RBC membranes are incubated in chloroform: methanol solution. Residual RBC membranes and proteins precipitates are removed by filtration. The extract comprised of glycolipids, phospholipids, and cholesterol is dried then sonicated in saline in a sonication bath to generate ~4.0 gm *α*-gal liposomes from 1.0 liter rabbit RBC (~10^15^ *α*-gal epitopes/mg). These liposomes are further sonicated by a sonication probe on ice, into submicroscopic *α*-gal nanoparticles which are filtered through 0.45 *μ*m filter and then through 0.2 *μ*m filter to ensure sterility [19, 21, 23]. The nanoparticles produced by this method have a wall of phospholipids and cholesterol in which *α*-gal glycolipids are anchored via the fatty acid tails of their ceramide portion (Figure 1). The illustrated glycolipid in Figure 1 has 10 sugar units in its carbohydrate chain and 2 branches (antennae), each capped with an *α*-gal epitope. The *α*-gal glycolipids originating from rabbit RBC membranes and presented on these *α*-gal nanoparticles are of various lengths ranging from 5 to 40 carbohydrate units and carrying 1–8 branches each capped with an *α*-gal epitope [50–55]. The *α*-nanoparticles are highly stable since they contain no tertiary structures and can be kept at 4°C or −20°C for several years without losing activity. This stability is indicated by the similarity in the ability of *α*-gal nanoparticles kept at 4°C for 4 years and that of freshly prepared *α*-gal nanoparticles to bind anti-Gal, as measured by ELISA (using *α*-gal nanoparticles as solid-phase Ag), and by the ability of the two *α*-gal nanoparticles preparations to activate serum complement following anti-Gal binding, as assayed in complement consumption assays (unpublished observations).

Based on the extensive in vitro interaction between anti-Gal and *α*-gal nanoparticles, it could be expected that, following topical application of these nanoparticles to wounds, they will readily bind the natural anti-Gal Ab that is released from ruptured blood vessels within the wound and is present in the fluid film in wounds. As schematically illustrated in Figure 1, this Ag/Ab interaction activates the complement system, generating chemotactic peptides that recruit macrophages which bind the anti-Gal coated nanoparticles and produce cytokines and growth factors that orchestrate the healing of wounds [19–21, 23]. The experiments demonstrating the various processes involved in *α*-gal nanoparticles mediated wound healing are described in sections below.

## 4. Experimental Animal Models for Studying Anti-Gal/***α***-gal Nanoparticles Interaction

In vivo studies on anti-Gal mediated effects of *α*-gal nanoparticles cannot be performed in standard experimental animal models since mice, rats, guinea-pigs, rabbits, and pigs (as well as other nonprimate mammals) all produce *α*-gal epitopes on their cells by the glycosylation enzyme *α*1,3galactosyltransferase (*α*1,3GT) [37, 38]. Therefore, nonprimate mammals cannot produce anti-Gal as they are immunotolerant to the *α*-gal epitope [37, 38, 56]. As indicated above, Old World monkeys, apes and humans are the only mammalian species producing anti-Gal since they lack the *α*-gal epitope [22, 27, 37]. However, in the recent two decades, several groups succeeded in generating *α*1,3GT knockout mice (GT-KO mice) [57, 58] and *α*1,3GT knockout pigs (GT-KO pigs) [59–61]. These knockout mice and pigs lack *α*-gal epitopes because of targeted disruption (knockout) of the *α*1,3GT gene, and thus they are not immunotolerant to it [19, 58, 62, 63]. Because GT-KO mice are usually kept in a clean environment they lack the gastrointestinal flora that induces production of the natural anti-Gal Ab and therefore, natural production of this Ab is usually low. However, these mice readily produce anti-Gal following few immunizations with xenograft tissues expressing multiple *α*-gal epitopes, such as pig kidney membrane homogenate [19]. In contrast, GT-KO pigs have the required natural flora and thus produce the natural anti-Gal Ab [62, 63]. Both GT-KO mice and GT-KO pigs were found to be suitable experimental models for studying the effects of *α*-gal nanoparticles on wound healing [19–21].

## 5. Recruitment of Macrophages by ***α***-gal Nanoparticles

As indicated in Figure 1, it is expected that the first event which occurs following application of *α*-gal nanoparticles to wounds is the interaction between serum anti-Gal and *α*-gal epitopes on these nanoparticles. This interaction leads to activation of the complement system (Step 1 in Figure 1). The in vivo activation of complement following anti-Gal/*α*-gal epitope interaction has been repeatedly demonstrated in xenotransplantation studies in which xenografts expressing *α*-gal epitopes on their endothelial cells underwent complement mediated hyperacute rejection following binding of the recipient's anti-Gal to xenograft *α*-gal epitopes [40–42]. Accordingly, no such hyperacute rejection has been observed when the xenograft was obtained from a GT-KO pig lacking *α*-gal epitopes [64–66]. Like any Ag/Ab interaction activating the complement cascade, also complement activation by anti-Gal binding to *α*-gal nanoparticles results in production of complement cleavage peptides including C5a and C3a. These complement cleavage peptides are among the most potent physiologic chemotactic factors (chemoattractants) which induce rapid recruitment of macrophages (Step 2 in Figure 1).

**Figure 2:**
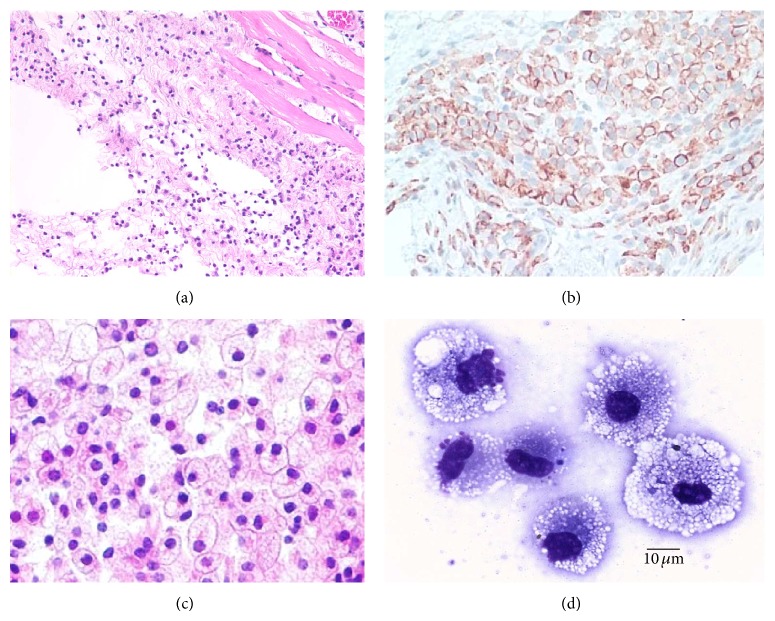
Recruitment of macrophages by 10 mg *α*-gal nanoparticles injected intradermal in anti-Gal producing GT-KO mice. (a) Macrophages migrating toward the injection site, 24 h after injection. The injection site is indicated as the empty area since the *α*-gal nanoparticles are dissolved by the alcohol fixative (H&E ×100). (b) The injection site after 4 days immunostained with macrophage specific HRP-anti-F4/80 Ab. The recruited cells are stained by this Ab (×200). (c) The injection site after 7 days demonstrating many large macrophages with ample cytoplasm (H&E ×400). (d) Individual macrophages isolated on day 7 displaying large size and multiple cytoplasmic vacuoles, probably the result of activation and uptake of the anti-Gal coated *α*-gal nanoparticles (Wright staining ×1000). Modified from [19].

Recruitment of macrophages could be demonstrated following intradermal injection of 10 mg *α*-gal nanoparticles in anti-Gal producing GT-KO mice. Extensive macrophage recruitment at the injection sites was observed already within 24 h after injection of the nanoparticles (Figure 2(a)). It should be noted that recruitment of neutrophils was observed within 12 h after injection [19]. However, after 24 h, most of the neutrophils disappeared and the recruited cells were primarily macrophages. By 48 h no neutrophils were found at the injection site, whereas the number of recruited macrophages further increased [19]. Immunostaining of the recruited cells on day 4 by the macrophage specific anti-F4/80 Ab indicated that most of these cells were indeed macrophages (Figure 2(b)). By day 7, the recruited cells were large and displayed ample cytoplasm, suggesting activation of the macrophages (Figure 2(c)). Individual isolated macrophages were found to be very large (20–30 *μ*m) and contained multiple vacuoles that represented the internalized *α*-gal nanoparticles (Figure 2(d)). The presence of multiple macrophages at the injection site was also observed on day 14 after injection [19]. However, by day 21 all macrophages disappeared and the skin displayed complete restoration of its normal structure with no indication of granuloma. This finding raises the possibility that all macrophages migrate away from the *α*-gal nanoparticles injection site after 3 weeks.

Intradermal injection of *α*-gal nanoparticles in wild-type (WT) mice (i.e., mice producing *α*-gal epitopes and lacking anti-Gal) did not induce recruitment of macrophages [19]. This implied that the observed recruitment of macrophages in GT-KO mice was dependent on the presence of anti-Gal which interacts with *α*-gal nanoparticles (Step 1 in Figure 1). The need for complement activation for recruitment of macrophages was further demonstrated by inhibition of the complement activation process. Intradermal injection of *α*-gal nanoparticles together with cobra venom factor (inhibitor of complement activation cascade) into GT-KO mice resulted in no recruitment of macrophages [19]. This finding strongly suggests that macrophage recruitment (Step 2 in Figure 1) is dependent on complement cleavage chemotactic factors formed as a result of complement activation by anti-Gal/*α*-gal nanoparticles interaction.

The recruited macrophages in GT-KO mice were further studied in biologically inert polyvinyl alcohol (PVA) sponge discs containing 10 mg *α*-gal liposomes (*α*-gal nanoparticles with size >1 *μ*m) and implanted subcutaneously. All recruited cells retrieved from these implanted PVA sponge discs 3, 6, or 9 days after implantation were stained with macrophage specific anti-CD11b and anti-CD14 Abs [20]. No recruited T cells or B cells were detected in the PVA sponge discs. The cells retrieved from the sponge discs on day 6 displayed the morphology of large macrophages with multiple cytoplasmic vacuoles that may represent internalized anti-Gal coated *α*-gal liposomes [20]. In absence of *α*-gal nanoparticles in implanted PVA sponge discs, recruitment of macrophages was ~90% lower than in presence of these nanoparticles [20].

## 6. Activation of the Recruited Macrophages by Fc/Fc***γ***R Interaction

**Figure 3:**
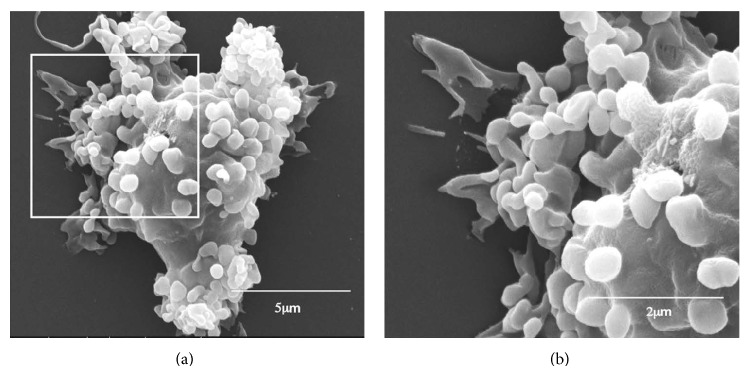
Scanning electron microscopy of anti-Gal coated *α*-gal nanoparticles binding to macrophages via Fc/Fc*γ*R interaction. *α*-gal nanoparticles coated with anti-Gal Ab were coincubated with adherent GT-KO pig macrophages for 2 hours at room temperature, washed to remove nonadherent nanoparticles, and processed for electron microscopy analysis. Many *α*-gal nanoparticles adhere to the surface of a representative macrophage. The inset in (a) is enlarged in (b). Modified from [23].

Step 3 in Figure 1 illustrates activation of the recruited macrophages reaching the *α*-gal nanoparticles as a result of interaction between the Fc “tails” of anti-Gal coating *α*-gal nanoparticles and Fc*γ*R on these macrophages. This Fc/Fc*γ*R interaction between anti-Gal coated *α*-gal nanoparticles and macrophages is demonstrated in Figure 3 where the nanoparticles coated with anti-Gal were incubated with GT-KO pig macrophages for 2 h. This coincubation resulted in attachment of multiple *α*-gal nanoparticles to the macrophages via the Fc/Fc*γ*R interaction. No significant binding of *α*-gal nanoparticles to macrophages was observed if the nanoparticles were not coated with anti-Gal [23]. A similar Fc/Fc*γ*R interaction was observed with *α*-gal nanoparticles coated with GT-KO mouse anti-Gal, incubated with mouse macrophages and analyzed for binding by flow cytometry [19].

The binding of *α*-gal nanoparticles to macrophages via Fc/Fc*γ*R interaction seemed to activate the macrophages. This is suggested by the large size of the macrophages observed subcutaneously 7 days after administration of the nanoparticles (Figures 2(c) and 2(d)). Activation of macrophages binding the *α*-gal nanoparticles is further indicated by the production of various “prohealing” cytokines/growth factors capable of accelerating wound healing, as hypothesized in Step 4 of Figure 1. VEGF is one of the pivotal cytokines in wound healing which induces vascularization of the healing wound. In vitro incubation for 24 h of GT-KO mouse macrophages with anti-Gal coated *α*-gal nanoparticles resulted in production of VEGF at a level that was twice as high as that secreted by macrophages in the absence of *α*-gal nanoparticles [19]. Incubation of macrophages with *α*-gal nanoparticles in the absence of anti-Gal resulted in VEGF production at a level similar to the control level produced by macrophages incubated without the nanoparticles [19].

**Figure 4:**
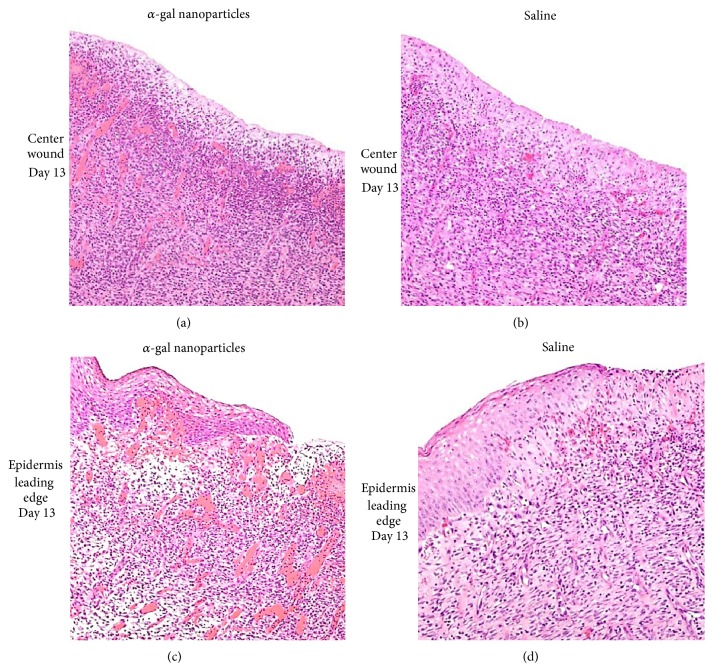
Vascularization of GT-KO pig wounds treated with *α*-gal nanoparticles (100 mg) or with saline and studied on day 13. The presented histology is of the centers of wound (not covered by regenerating epidermis), or wound areas under the leading edge of regenerating epidermis treated with *α*-gal nanoparticles (a and c, resp.) or with saline (b and d, resp.). There are many more macrophages and blood vessels (filled with red stained RBC) in wound treated with *α*-gal nanoparticles than in those treated with saline. Representative wounds from 6 GT-KO pigs (H&E ×200). Modified from [21].

The increased production of VEGF by activated macrophages within wounds is further suggested by the extensive vascularization of wounds in GT-KO pigs treated with *α*-gal nanoparticles (Figure 4). As further detailed in the section on GT-KO pig wound healing below, wounds on the back of those pigs were 20 × 20 mm and ~3 mm deep. The wounds were treated by topical application of *α*-gal nanoparticles or saline and covered with dressing which was replaced every 3-4 days [21]. As expected, the day 13 granulation tissue in wounds treated with *α*-gal nanoparticles contained many more macrophages than that in saline treated wounds (Figure 4). In addition, the day 13 granulation tissue in the *α*-gal nanoparticles treated wounds (Figures 4(a) and 4(c)) displayed a much higher concentration of blood vessels than that in saline treated wounds of the same GT-KO pig (Figures 4(b) and 4(d)). This higher vascularization may reflect the increased production of VEGF in *α*-gal nanoparticles treated wounds because of activation of recruited macrophages by anti-Gal coated *α*-gal nanoparticles that interact with Fc*γ*R of the macrophages (Step 4 in Figure 1).

An alternative approach for measuring in vivo production of various cytokines/growth factors was quantitative real time (RT) PCR for mRNA level of such cytokines within skin of GT-KO mice injected with *α*-gal nanoparticles. Such analysis demonstrated increased production of FGF, IL1, PDGF, and CSF in comparison to GT-KO mouse skin injected with nanoparticles lacking *α*-gal epitopes [19]. These findings further support the assumption that recruited macrophages undergoing Fc/Fc*γ*R interaction with anti-Gal coated *α*-gal nanoparticles are activated to produce and secrete cytokines/growth factors that promote repair and regeneration of injured tissues. The recruitment of stem cells by the cytokines/growth factors secreted from these activated macrophages (Step 5 in Figure 1) was studied in PVA sponge discs containing porcine meniscus cartilage extracellular matrix (ECM) homogenate mixed with *α*-gal nanoparticles. Such PVA sponge discs were implanted subcutaneously for 5 weeks in anti-Gal producing GT-KO mice [67]. Demonstration of meniscus like fibrocartilage generation in such sponge discs suggested that stem cells recruited into these PVA sponge discs by secretions from activated macrophages were “instructed” by the meniscus cartilage ECM fragments to differentiate into fibrochondroblasts that produce fibrocartilage [67].

## 7. Treatment of Wounds with ***α***-gal Nanoparticles in GT-KO Mice

The effect of *α*-gal nanoparticles treatment on the healing of skin wounds was first studied in anti-Gal producing GT-KO mice. Oval-shaped excisional deep skin wounds with the size of ~3 × 6 mm were formed under anesthesia in the right abdominal flank of the mice. The wounds were covered with spot bandage coated with 10 mg *α*-gal nanoparticles or with 10 mg nanoparticles lacking *α*-gal epitopes (prepared from GT-KO pig RBC). Control wounds were covered with spot bandages containing saline. Wounds treated with *α*-gal nanoparticles displayed 95–100% healing (i.e., most or all the wound surface area was covered with regrown epidermis) within 6 days after treatment [19]. In contrast, wounds treated with nanoparticles lacking *α*-gal epitopes or those treated with saline displayed only marginal regrowth of the epidermis which covered <20% of the wound surface at day 6 after treatment [19]. The wounds treated with nanoparticles lacking *α*-gal epitopes or with saline displayed 95–100% healing only by days 12–14. These studies indicated that the treatment of GT-KO mouse wounds with *α*-gal nanoparticles decreased the healing time by >50% in comparison to control wounds treated with saline or with nanoparticles lacking *α*-gal epitopes. Histological evaluation of wounds further indicated that the processes of vascularization, fibroblast migration, and collagen deposition in the dermis also are accelerated in wounds treated with *α*-gal nanoparticles in comparison to saline treated wounds [19]. Studies performed with *α*-gal liposomes (*α*-gal nanoparticles with size >1 *μ*m) were also found to induce acceleration of wound healing. However, *α*-gal nanoparticles formed following extensive sonication of *α*-gal liposomes were found to be somewhat more effective in accelerating wound healing than comparable amounts of liposomes. This improved healing of the submicroscopic nanoparticles is possibly because of better dispersion throughout the wound [19].

In studies with *α*-gal liposomes which preceded those with *α*-gal nanoparticles, these liposomes were also found to accelerate healing of burns in GT-KO mice. Small thermal injuries (2 × 3 mm) were performed in the shaved skin of anesthetized anti-Gal producing GT-KO mice by a brief touch of a heated metal spatula. Such burns are comparable to second degree burns in humans in that the epidermis and part of the dermis are damaged by the thermal injury. The burns were covered with spot bandages coated with 10 mg *α*-gal liposomes, or with saline as control [20]. Burns treated with *α*-gal liposomes displayed a much faster recruitment of neutrophils and macrophages than those treated with saline. Moreover, *α*-gal liposomes treated burns were covered with regenerating epidermis, including* Stratum corneum* by day 6 after treatment, whereas saline treated wounds displayed similar healing only after ~12 days [20]. No acceleration of burn healing was observed in wild-type mice (lacking anti-Gal) treated with *α*-gal liposomes in comparison to burns treated with saline, further implying that the accelerated healing process occurs only in the presence of the anti-Gal Ab and thus, is dependent on anti-Gal/*α*-gal epitopes interaction.

## 8. Prevention of Scar Formation Following Treatment with ***α***-gal Nanoparticles

Small wounds usually heal fast and restore the original structure and cellular composition of the tissue. However, injuries of large size are slow to heal because of the extensive vascularization and the high number of cells required for repopulation of the injured tissue. Under such circumstances the default mechanism of fibrosis replaces the slower repair and regeneration processes. This fibrosis consists of formation of dense connective tissue and low level of vascularization, resulting in scar formation. The fibrotic scar serves as barrier between pathogens in the external environment and inner tissues. Thus, it was of interest to determine whether wounds treated with *α*-gal nanoparticles display fibrosis and scar formation similar to that observed in saline treated wounds.

**Figure 5:**
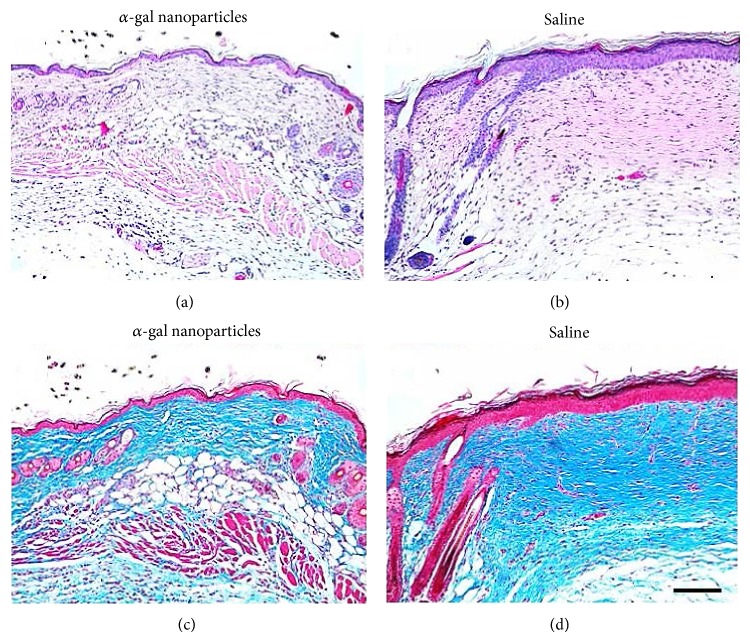
Prevention of scar formation in GT-KO mouse wounds treated with *α*-gal nanoparticles. Representative wound treated for 28 days with *α*-gal nanoparticles and stained with H&E (a) and with trichrome (c). Representative saline treated wound after 28 days, stained with H&E (b) and with trichrome (d). Collagen is stained blue by trichrome. *α*-gal nanoparticles treated wounds (a, c) display restoration of normal skin structure, including thin epidermis, loose connective tissue in the dermis and appearance of skin appendages including hair, sebaceous glands, fat and muscle cells in the hypodermis. Saline treated wounds (b, d) undergo fibrosis to form a scar characterized by dense connective tissue as a result of extensive collagen secretion by multiple infiltrating fibroblasts, no skin appendages and hyperplastic epidermis. The hair shafts in the left are of the uninjured area bordering the wound. Scale bar in (d) is 100 *μ*m (×100). Specimens are representative of 5 mice per group. Modified from [19].

Wounds of GT-KO mice treated with saline and inspected 28 days after treatment displayed a large area of dense fibrotic dermis devoid of skin appendages and a distinct epidermal hyperplasia (≥5 layers of cells), both characteristic to scar formation (Figures 5(b) and 5(d)) [19]. In contrast, *α*-gal nanoparticle treated wounds inspected after 28 days displayed dermis with normal density of collagen and epidermis of normal 2-cell layers thickness (Figures 5(a) and 5(b)). Moreover, *α*-gal nanoparticles treated wounds displayed in the dermis regenerating appendages such as hair follicles and sebaceous glands, as well as fat cells and muscle cells. No granuloma, macrophages, or keloids were observed in *α*-gal nanoparticles treated wounds or in control saline treated wounds at 28 days [19]. It is probable that this absence of scar tissue in wounds treated with *α*-gal nanoparticles is the result of the accelerated repair and regeneration process induced by anti-Gal interaction with the nanoparticles. This acceleration of the healing process is likely to result in restoration of normal cellular skin components in the wound prior to the onset of the fibrosis process; thus scar formation is avoided.

## 9. Accelerating Wound Healing in GT-KO Pigs

As indicated above, there are only two nonprimate mammalian experimental models that lack the *α*-gal epitope and produce the anti-Gal Ab, GT-KO mice, and GT-KO pigs. Whereas anti-Gal production has to be induced in GT-KO mice [19, 45], GT-KO pigs naturally produce anti-Gal [62, 63]. Thus, it was of interest to determine whether the acceleration of wound healing observed in GT-KO mice can be validated in the large animal model of GT-KO pig in which the skin structure is very similar to that of human skin.

**Figure 6:**
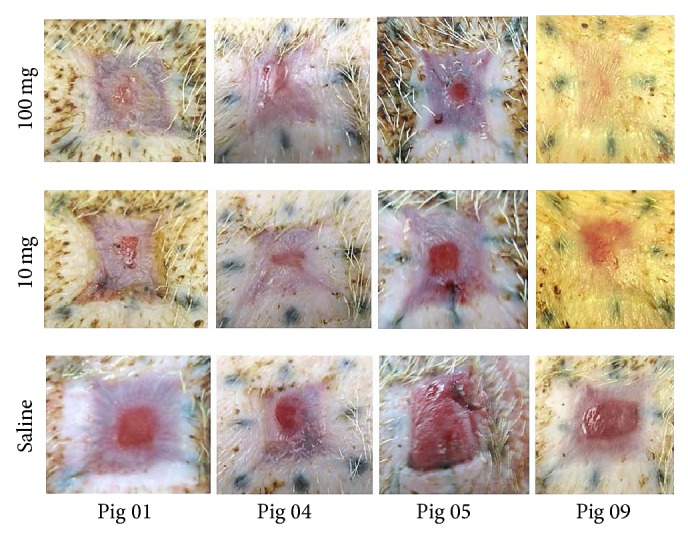
Healing of excisional wounds in the skin of GT-KO pigs treated with *α*-gal nanoparticles or with saline and viewed on day 13. The original size of the wounds was 20 × 20 mm and 3 mm deep. The border of wounds was marked by tattooed dots to determine contraction during healing. Control wounds treated with saline display partial regeneration of the epidermis as a result of physiologic healing. However, wounds treated with 100 mg *α*-gal nanoparticles are almost completely or completely covered with regenerating epidermis. Wounds treated with 10 mg *α*-gal nanoparticles heal faster than saline treated wounds, however, somewhat slower than wounds treated with 100 mg *α*-gal nanoparticles in the same pig. There are no significant differences of wound contractions between saline treated and *α*-gal nanoparticles treated wounds. Modified from [21].

Eight excisional 20 × 20 mm square wounds (~3 mm deep) were formed on the back of 3-month-old GT-KO pigs. Borders of the wounds were marked by tattooed dots prior to wounding in order to evaluate wound contraction. The wounds in each GT-KO pig were treated by topical application of 100 mg *α*-gal nanoparticles in 1.0 mL (4 wounds), 10 mg *α*-gal nanoparticles (2 wounds), or saline (2 wounds) and covered with dressings that were changed every 3-4 days [21]. On day 7, all wounds were filled with equal amounts of granulation tissue; however, the concentration of macrophages was found to be higher in *α*-gal nanoparticles treated wounds than in saline treated wounds [21]. Wound size was not significantly different on day 7 in wounds treated with *α*-gal nanoparticles or saline. Distinct differences in wound healing were observed on day 13 (Figure 6). Wounds treated with 100 mg *α*-gal nanoparticles were completely or almost completely covered with regenerating epidermis, whereas saline treated wounds displayed only partial healing. Complete regeneration of epidermis in saline treated wounds was observed only 18–22 days after wounding. On day 13, the area not covered by regenerating epidermis in wounds treated with 100 mg *α*-gal nanoparticles was 10-fold smaller than that in saline treated wounds (Figure 6) [21]. Moreover, the extent of angiogenesis was much higher in *α*-gal nanoparticles treated wounds than in saline treated wounds (Figures 4(a) and 4(c) versus Figures 4(b) and 4(d), resp.). No significant differences were observed, however, in wound contraction (marked by conversion of tattooed dots into stretched lines) in *α*-gal nanoparticles and saline treated wounds (Figure 6). There seemed to have been a dose response in the healing effect of *α*-gal nanoparticles. Healing of wounds treated with 10 mg *α*-gal nanoparticles was slower than that observed in wounds treated with 100 mg *α*-gal nanoparticles but faster than that in saline treated wounds (Figure 6) [21]. Healed wounds inspected 60 days after injury displayed no keloids formation under any of the treatments. The *α*-gal nanoparticles treated wounds also displayed hair growth [21]. No scar formation was observed in both *α*-gal nanoparticles and saline treated wounds.

Overall, these observations on accelerated healing of wounds treated with *α*-gal nanoparticles in GT-KO pigs and GT-KO mice, growth of skin appendages, absence of scar tissue in GT-KO mice, and absence of keloids in both pig and mouse experimental models, all suggest that similar treatment in humans may induce accelerated wound healing without adverse effects.

## 10. Methods for Application of ***α***-gal Nanoparticles

Because the *α*-gal nanoparticles require interaction with the natural anti-Gal Ab for the induction of accelerated healing of wound, they are likely to be effective in “wet” wounds or burns. The fluid films in such wounds are formed by plasma containing anti-Gal and complement proteins leaking from injured capillaries. In contrast, dry wounds covered with a scab do not enable the *α*-gal nanoparticles to interact with anti-Gal and to activate the complement cascade; therefore these nanoparticles may have no beneficial effects in such wounds.

As mentioned above, *α*-gal nanoparticles were found to display high stability during storage at 4°C or −20°C for at least 4 years, as indicated by conserving their ability to bind the anti-Gal Ab and activate serum complement even after such prolonged storage (unpublished observations). This suggests that *α*-gal nanoparticles may be applied to wounds and burns by a variety of methods and wound care devices. In addition to direct application of *α*-gal nanoparticles as suspension, these nanoparticles may be stored in a dried form on wound dressing and then applied to the wound as part of the dressing. *α*-gal nanoparticles may also be applied to large areas of wounds and burns in an aerosol form (i.e., stored as a suspension under pressure in a container that can spray the suspension in an aerosolized form), or as foam. These nanoparticles may further be incorporated into biodegradable scaffold materials such as natural or recombinant collagen. Dressings that include collagen sheets are used for treatment of large wounds and burns. Since collagen enables effective diffusion of complement proteins and immunoglobulins from the fluid film of the wound, anti-Gal/*α*-gal nanoparticles interaction may occur within collagen sheets placed on skin injuries. In addition, nanoparticles may diffuse from the collagen sheet into the wound. The ensuing complement activation will generate chemotactic factors that recruit macrophages into the treated injuries and accelerate healing in these injuries.

## 11. Future Directions: ***α***-gal Nanoparticles Treatment of Internal Injuries

Healing of internal injuries is mediated by mechanisms similar to those in skin injuries and involves recruitment and activation of macrophages as a prerequisite for repair and regeneration [68–70]. Thus, *α*-gal nanoparticles may also accelerate healing of various internal injuries, thereby restoring the original structure and function of the injured tissue and avoiding scar formation. Three of the possible uses of *α*-gal nanoparticles in treatments of internal injuries, which may be of interest to study, are as follows.

### 11.1. Healing of Surgical Incisions

Surgical incisions, their suturing, and the resulting internal injuries represent a major part of the morbidity of the abdominal surgery. Administration of *α*-gal nanoparticles to such injuries may accelerate their healing as in skin injuries. In order to ensure retention of the nanoparticles at the site of their administration *α*-gal nanoparticles should be introduced in semisolid biodegradable “fillers” such as hydrogel or fibrin glue. Diffusion of anti-Gal and complement within such fillers is likely to result in interaction with the *α*-gal nanoparticles and activation of the healing process described in Figure 1. Similarly, collagen sheets or other biodegradable scaffolds containing *α*-gal nanoparticles and applied together with a surgical mesh may accelerate the healing of the abdominal wall following hernia surgery.

### 11.2. Regeneration of Postinfarction Ischemic Myocardium

In myocardial infarction, cardiomyocytes in the ischemic area die, resulting in injury of the myocardium. As in wound healing, macrophages migrate to the injured myocardium, debride it of dead cells, and secrete cytokines/growth factors that induce angiogenesis and recruitment of mesenchymal stem cells or of myocardium progenitor cells from uninjured areas of the heart (cf. [71, 72]). If the size of the injury is small, these stem and progenitor cells are instructed by the ECM to differentiate into cardiomyocytes that repopulate the tissue and restore its physiologic activity. However, if the ischemic area is large, the outcome of the healing process is fibrosis which occurs faster than the regenerative process, resulting in irreversible scar formation and impairment of myocardium activity. It is possible to inject *α*-gal nanoparticles into the ischemic myocardium by a catheter navigated into the left ventricle. Such injection will result in rapid and extensive recruitment of macrophages, as previously shown in ischemic myocardium of GT-KO mice [23]. Activation of the recruited macrophages by interaction with anti-Gal coated *α*-gal nanoparticles may induce angiogenesis and effective recruitment of stem cells which, in turn, will be instructed by the ECM to differentiate into cardiomyocytes that repopulate and regenerate the injured tissue. In analogy with prevention of scar formation in wounds (Figure 5) it is possible that the regeneration of the injured myocardium because of the accelerated healing by *α*-gal nanoparticles may occur before the onset of fibrosis.

### 11.3. Regeneration of Injured Nerves

Regeneration of nerves requires growth of multiple sprouts from the injured axons. The nerve can regenerate if one of the sprouts “succeeds” in growing across the lesion and penetrating into the distal axonal tube. This regrowth of an axon sprout into the distal axonal tube can occur only within a given period of time after injury since the process of fibrosis of the nerve lesion is also initiated shortly after the injury. Thus, if sprouts of the severed axons fail to “find” the distal axonal tubes and grow into them, the default fibrosis process will “take over,” resulting in formation of dense connective tissue in the lesion area. Such fibrosis will block further growth of sprouts into distal axonal tubes. The extent of sprout growth in injured nerves is dependent on the angiogenesis within lesion sites since the axonal sprouts grow along de novo formed capillaries [73]. Formation of capillaries is dependent on VEGF secretion by macrophages recruited into the nerve lesion site. This process of axonal regrowth may be amplified by *α*-gal nanoparticles. As illustrated in Figure 1, anti-Gal binding to *α*-gal nanoparticles results in complement activation, rapid chemotactic recruitment of macrophages, and activation of these macrophages to secrete a variety of cytokines/growth factors including VEGF [19]. It is therefore possible that application of *α*-gal nanoparticles to nerve lesion sites in spinal cord or in peripheral nerve injuries shortly after the injury will induce rapid recruitment of macrophages. The recruited macrophages will be further activated to secret VEGF into the lesion and induce angiogenesis resulting in formation of an extensive capillaries network. These multiple capillaries, in turn, will enable growth of many axonal sprouts, thereby increasing the probability that some of these sprouts will “find” the distal axonal tubes and thus, will regenerate the injured nerve.

## 12. Conclusions

Wound healing therapy by *α*-gal nanoparticles harnesses the immunological potential of the natural anti-Gal Ab which is the most abundant Ab in humans. Application of *α*-gal nanoparticles to wounds results in binding of anti-Gal to the multiple *α*-gal epitopes on these nanoparticles. This Ag/Ab interaction activates the complement system and generates complement cleavage chemotactic factors that induce rapid recruitment of macrophages. The recruited macrophages interact with the anti-Gal coating *α*-gal nanoparticles via their Fc*γ*R. Such macrophages are activated to secrete cytokines/growth factors that mediate healing and recruit stem cells. In GT-KO mice and GT-KO pigs, *α*-gal nanoparticles reduce the healing time of wounds and burns by 40–60% and decrease or eliminate scar formation without formation of keloids. Since the mechanism for repair and regeneration is the same for external and internal injuries, it is possible that administration of *α*-gal nanoparticles to internal injuries such as wounds associated with surgical incisions, postinfarction ischemic myocardium, and nerve injuries may induce appropriate healing of the injured tissues, instead of fibrosis and scar formation.

## Abbreviations

*α*-gal epitope: Gal*α*1-3Gal*β*1-4GlcNAc-R epitope
*α*1,3GT: *α*1,3galactosyltransferase
*α*-gal liposomes: Liposomes presenting multiple *α*-gal epitopes
*α*-gal nanoparticles: Submicroscopic liposomes with glycolipids that carry multiple *α*-gal epitopes
ECM: Extracellular matrix
GT-KO mice or pigs: Knockout mice or pigs for the *α*1,3galactosyltransferase gene
PVA sponge discs: Polyvinyl alcohol sponge discs
RBC: Red blood cells
VEGF: Vascular endothelial growth factor.

## References

[1] Sen C. K., Gordillo G. M., Roy S. (2009). Human skin wounds: a major and snowballing threat to public health and the economy. *Wound Repair and Regeneration*.

[2] Leibovich S. J., Ross R. (1975). The role of the macrophage in wound repair. A study with hydrocortisone and anti-macrophage serum. *American Journal of Pathology*.

[3] DiPietro L. A. (1995). Wound healing: the role of the macrophage and other immune cells. *Shock*.

[4] Singer A. J., Clark R. A. F. (1999). Cutaneous wound healing. *The New England Journal of Medicine*.

[5] Martin P. (1997). Wound healing—aiming for perfect skin regeneration. *Science*.

[6] Martin P., Leibovich S. J. (2005). Inflammatory cells during wound repair: the good, the bad and the ugly. *Trends in Cell Biology*.

[7] Franz M. G., Steed D. L., Robson M. C. (2007). Optimizing healing of the acute wound by minimizing complications. *Current Problems in Surgery*.

[8] Gurtner G. C., Werner S., Barrandon Y., Longaker M. T. (2008). Wound repair and regeneration. *Nature*.

[9] Sica A., Mantovani A. (2012). Macrophage plasticity and polarization: in vivo veritas. *Journal of Clinical Investigation*.

[10] Piccolo M.-T. S., Wang Y., Sannomiya P. (1999). Chemotactic mediator requirements in lung injury following skin burns in rats. *Experimental and Molecular Pathology*.

[11] Shukaliak J. A., Dorovini-Zis K. (2000). Expression of the *β*-chemokines RANTES and MIP-1*β* by human brain microvessel endothelial cells in primary culture. *Journal of Neuropathology and Experimental Neurology*.

[12] Low Q. E. H., Drugea I. A., Duffner L. A. (2001). Wound healing in MIP-1*α*(−/−) and MCP-1(−/−) mice. *The American Journal of Pathology*.

[13] Wood G. W., Hausmann E., Choudhuri R. (1997). Relative role of CSF-1, MCP-1/JE, and RANTES in macrophage recruitment during successful pregnancy. *Molecular Reproduction and Development*.

[14] Heinrich S. A., Messingham K. A. N., Gregory M. S. (2003). Elevated monocyte chemoattractant protein-1 levels following thermal injury precede monocyte recruitment to the wound site and are controlled, in part, by tumor necrosis factor-*α*. *Wound Repair and Regeneration*.

[15] Shallo H., Plackett T. P., Heinrich S. A., Kovacs E. J. (2003). Monocyte chemoattractant protein-1 (MCP-1) and macrophage infiltration into the skin after burn injury in aged mice. *Burns*.

[16] Snyderman R., Pike M. C. (1984). Chemoattractant receptors on phagocytic cells. *Annual Review of Immunology*.

[17] Damerau B. (1987). Biological activities of complement-derived peptides. *Reviews of Physiology, Biochemistry and Pharmacology*.

[18] Haeney M. R. (1998). The role of the complement cascade in sepsis. *Journal of Antimicrobial Chemotherapy*.

[19] Wigglesworth K. M., Racki W. J., Mishra R., Szomolanyi-Tsuda E., Greiner D. L., Galili U. (2011). Rapid recruitment and activation of macrophages by anti-gal/*α*-gal liposome interaction accelerates wound healing. *The Journal of Immunology*.

[20] Galili U., Wigglesworth K., Abdel-Motal U. M. (2010). Accelerated healing of skin burns by anti-Gal/*α*-gal liposomes interaction. *Burns*.

[21] Hurwitz Z. M., Ignotz R., Lalikos J. F., Galili U. (2012). Accelerated porcine wound healing after treatment with *α*-gal nanoparticles. *Plastic & Reconstructive Surgery*.

[22] Galili U., Rachmilewitz E. A., Peleg A., Flechner I. (1984). A unique natural human IgG antibody with anti-*α*-galactosyl specificity. *Journal of Experimental Medicine*.

[23] Galili U. (2013). Macrophages recruitment and activation by *α*-gal nanoparticles accelerate regeneration and can improve biomaterials efficacy in tissue engineering. *The Open Tissue Engineering and Regenerative Medicine Journal*.

[24] Sandrin M. S., Vaughan H. A., Dabkowski P. L., McKenzie I. F. C. (1993). Anti-pig IgM antibodies in human serum react predominantly with Gal(*α* 1—3)Gal epitopes. *Proceedings of the National Academy of Sciences of the United States of America*.

[25] Hamadeh R. M., Galili U., Zhou P., Griffiss J. M. (1995). Anti-*α*-galactosyl immunoglobulin A (IgA), IgG, and IgM in human secretions. *Clinical and Diagnostic Laboratory Immunology*.

[26] Holzknecht Z. E., Platt J. L. (1995). Identification of porcine endothelial cell membrane antigens recognized by human xenoreactive natural antibodies. *The Journal of Immunology*.

[27] Teranishi K., Manez R., Awwad M., Cooper D. K. C. (2002). Anti-Gal*α*1–3Gal IgM and IgG antibody levels in sera of humans and old world non-human primates. *Xenotransplantation*.

[28] Galili U., Macher B. A., Buehler J., Shohet S. B. (1985). Human natural anti-*α*-galactosyl IgG. II. The specific recognition of *α*(1 → 3)-linked galactose residues. *Journal of Experimental Medicine*.

[29] Galili U., Mandrell R. E., Hamadeh R. M., Shohet S. B., Griffiss J. M. (1988). Interaction between human natural anti-*α*-galactosyl immunoglobulin G and bacteria of the human flora. *Infection and Immunity*.

[30] Galili U., Anaraki F., Thall A., Hill-Black C., Radic M. (1993). One percent of human circulating B lymphocytes are capable of producing the natural anti-Gal antibody. *Blood*.

[31] Good A. H., Cooper D. K. C., Malcolm A. J. (1992). Identification of carbohydrate structures which bind human anti-porcine antibodies: implication for discordant xenografting in man. *Transplantation Proceedings*.

[32] Bennet W., Björkland A., Sundberg B. (2000). A comparison of fetal and adult porcine islets with regard to Gal *α* (1,3)Gal expression and the role of human immunoglobulins and complement in islet cell cytotoxicity. *Transplantation*.

[33] Baumann B. C., Stussi G., Huggel K., Rieben R., Seebach J. D. (2007). Reactivity of human natural antibodies to endothelial cells from Gal*α*(1,3)Gal-deficient pigs. *Transplantation*.

[34] Repik P. M., Strizki J. M., Galili U. (1994). Differential host-dependent expression of *α*-galactosyl epitopes on viral glycoproteins: a study of eastern equine encephalitis virus as a model. *Journal of General Virology*.

[35] Takeuchi Y., Porter C. D., Strahan K. M. (1996). Sensitization of cells and retroviruses to human serum by (*α*1–3) galactosyltransferase. *Nature*.

[36] Welsh R. M., O'Donnell C. L., Reed D. J., Rother R. P. (1998). Evaluation of the Gal*α*1–3Gal epitope as a host modification factor eliciting natural humoral immunity to enveloped viruses. *Journal of Virology*.

[37] Galili U., Clark M. R., Shohet S. B., Buehler J., Macher B. A. (1987). Evolutionary relationship between the natural anti-Gal antibody and the Gal*α*1—3Gal epitope in primates. *Proceedings of the National Academy of Sciences of the United States of America*.

[38] Galili U., Shohet S. B., Kobrin E., Stults C. L. M., Macher B. A. (1988). Man, apes, and Old World monkeys differ from other mammals in the expression of alpha-galactosyl epitopes on nucleated cells. *The Journal of Biological Chemistry*.

[39] Galili U. (1993). Interaction of the natural anti-Gal antibody with *α*-galactosyl epitopes: a major obstacle for xenotransplantation in humans. *Immunology Today*.

[40] Simon P. M., Neethling F. A., Taniguchi S. (1998). Intravenous infusion of Gal*α*1–3Gal oligosaccharides in baboons delays hyperacute rejection of porcine heart xenografts. *Transplantation*.

[41] Collins B. H., Cotterell A. H., McCurry K. R. (1995). Cardiac xenografts between primate species provide evidence for the importance of the *α*-galactosyl determinant in hyperacute rejection. *The Journal of Immunology*.

[42] Xu Y., Lorf T., Sablinski T. (1998). Removal of anti-porcine natural antibodies from human and nonhuman primate plasma in vitro and in vivo by a Gal*α*1-3Gal*β*1-4*β*Glc-X immunoaffinity column. *Transplantation*.

[43] Watier H., Guillaumin J.-M., Vallée I. (1996). Human NK cell-mediated direct and IgG-dependent cytotoxicity against xenogeneic porcine endothelial cells. *Transplant Immunology*.

[44] Kumagai-Braesch M., Satake M., Qian Y., Holgersson J., Möller E. (1998). Human NK cell and ADCC reactivity against xenogeneic porcine target cells including fetal porcine islet cells. *Xenotransplantation*.

[45] LaTemple D. C., Abrams J. T., Zhang S. Y., Galili U. (1999). Increased immunogenicity of tumor vaccines complexed with anti-Gal: studies in knockout mice for *α*1,3galactosyltransferase. *Cancer Research*.

[46] Rossi G. R., Mautino M. R., Unfer R. C., Seregina T. M., Vahanian N., Link C. J. (2005). Effective treatment of preexisting melanoma with whole cell vaccines expressing *α*(1,3)-galactosyl epitopes. *Cancer Research*.

[47] Galili U., Wigglesworth K., Abdel-Motal U. M. (2007). Intratumoral injection of *α*-gal glycolipids induces xenograft-like destruction and conversion of lesions into endogenous vaccines. *The Journal of Immunology*.

[48] Abdel-Motal U. M., Guay H. M., Wigglesworth K., Welsh R. M., Galili U. (2007). Increased immunogenicity of influenza virus vaccine by anti-Gal mediated targeting to antigen presenting cells. *Journal of Virology*.

[49] Abdel-Motal U., Wang S., Lu S., Wigglesworth K., Galili U. (2006). Increased immunogenicity of human immunodeficiency virus gp120 engineered to express Gal*α*1-3Gal*β*1-4GlcNAc-R epitopes. *Journal of Virology*.

[50] Eto T., Iichikawa Y., Nishimura K., Ando S., Yamakawa T. (1968). Chemistry of lipids of the posthemolytic residue or stroma of erythrocytes. XVI. Occurance of ceramide pentasaccharide in the membrane of erythrocytes and reticulocytes in rabbit. *Journal of Biochemistry*.

[51] Stellner K., Saito H., Hakomori S. (1973). Determination of aminosugar linkage in glycolipids by methylation. Aminosugar linkage of ceramide pentasaccharides of rabbit erythrocytes and of Forssman antigen. *Archives of Biochemistry and Biophysics*.

[52] Dabrowski U., Hanfland P., Egge H., Kuhn S., Dabrowski J. (1984). Immunochemistry of I/i-active oligo- and polyglycosylceramides from rabbit erythrocyte membranes. Determination of branching patterns of a ceramide pentadecasaccharide by 1H nuclear magnetic resonance. *Journal of Biological Chemistry*.

[53] Egge H., Kordowicz M., Peter-Katalinic J., Hanfland P. (1985). Immunochemistry of I/i-active oligo- and polyglycosylceramides from rabbit erythrocyte membranes. Characterization of linear, di-, and triantennary neolactoglycosphingolipids. *The Journal of Biological Chemistry*.

[54] Hanfland P., Kordowicz M., Peter-Katalinić J., Egge H., Dabrowski J., Dabrowski U. (1988). Structure elucidation of blood group B-like and I-active ceramide eicosa- and pentacosa-saccharides from rabbit erythrocyte membranes by combined gas chromatography-mass spectrometry; electron-impact and fast-atom-bombardment mass spectrometry; and two-dimensional correlated, relayed-coherence transfer, and nuclear overhauser effect 500-MHz 1H-N.m.r. spectroscopy. *Carbohydrate Research*.

[55] Honma K., Manabe H., Tomita M., Hamada A. (1981). Isolation and partial structural characterization of macroglycolipid from rabbit erythrocyte membranes. *Journal of Biochemistry*.

[56] Galili U. (2013). Anti-Gal: an abundant human natural antibody of multiple pathogeneses and clinical benefits. *Immunology*.

[57] Thall A. D., Maly P., Lowe J. B. (1995). Oocyte Gal*α*1–3Gal epitopes implicated in sperm adhesion to the zona pellucida glycoprotein ZP3 are not required for fertilization in the mouse. *Journal of Biological Chemistry*.

[58] Tearle R. G., Tange M. J., Zannettino Z. L. (1996). The *α*-1,3-galactosyltransferase knockout mouse: implications for xenotransplantation. *Transplantation*.

[59] Lai L., Kolber-Simonds D., Park K.-W. (2002). Production of *α*-1,3-galactosyltransferase knockout pigs by nuclear transfer cloning. *Science*.

[60] Phelps C. J., Koike C., Vaught T. D. (2003). Production of *α*1,3-galactosyltransferase-deficient pigs. *Science*.

[61] Kolber-Simonds D., Lai L., Watt S. R. (2004). Production of *α*-1,3-galactosyltransferase null pigs by means of nuclear transfer with fibroblasts bearing loss of heterozygosity mutations. *Proceedings of the National Academy of Sciences of the United States of America*.

[62] Dor F. J. M. F., Tseng Y.-L., Cheng J. (2004). *α*1-3-galactosyltransferase gene-knockout miniature swine produce natural cytotoxic anti-gal antobodies. *Transplantation*.

[63] Fang J., Walters A., Hara H. (2012). Anti-gal antibodies in *α*1,3-galactosyltransferase gene-knockout pigs. *Xenotransplantation*.

[64] Yamada K., Yazawa K., Shimizu A. (2005). Marked prolongation of porcine renal xenograft survival in baboons through the use of *α*1,3-galactosyltransferase gene-knockout donors and the cotransplantation of vascularized thymic tissue. *Nature Medicine*.

[65] Chen G., Qian H., Starzl T. (2005). Acute rejection is associated with antibodies to non-Gal antigens in baboons using Gal-knockout pig kidneys. *Nature Medicine*.

[66] Tseng Y.-L., Kuwaki K., Dor F. J. M. F. (2005). *α*1,3-galactosyltransferase gene-knockout pig heart transplantation in baboons with survival approaching 6 months. *Transplantation*.

[67] Galili U. (2015). Avoiding detrimental human immune response against mammalian extracellular matrix implants. *Tissue Engineering Part B: Reviews*.

[68] van Amerongen M. J., Harmsen M. C., van Rooijen N., Petersen A. H., Van Luyn M. J. A. (2007). Macrophage depletion impairs wound healing and increases left ventricular remodeling after myocardial injury in mice. *The American Journal of Pathology*.

[69] Mokarram N., Merchant A., Mukhatyar V., Patel G., Bellamkonda R. V. (2012). Effect of modulating macrophage phenotype on peripheral nerve repair. *Biomaterials*.

[70] Chazaud B., Brigitte M., Yacoub-Youssef H. (2009). Dual and beneficial roles of macrophages during skeletal muscle regeneration. *Exercise and Sport Sciences Reviews*.

[71] Radisic M., Christman K. L. (2013). Materials science and tissue engineering: repairing the heart. *Mayo Clinic Proceedings*.

[72] Bayomy A. F., Bauer M., Qiu Y., Liao R. (2012). Regeneration in heart disease—is ECM the key?. *Life Sciences*.

[73] Dray C., Rougon G., Debarbieux F. (2009). Quantitative analysis by in vivo imaging of the dynamics of vascular and axonal networks in injured mouse spinal cord. *Proceedings of the National Academy of Sciences of the United States of America*.

